# Can we use artificial intelligence to identify patients with Alzheimer's as defined by its biological markers by analysing brain scans?

**DOI:** 10.1002/alz70856_101507

**Published:** 2025-12-24

**Authors:** Sukhman Singh, Sofia Michopoulou, Xiaoxiao Li

**Affiliations:** ^1^ Faculty of Medicine, University of Southampton, Southampton, United Kingdom; ^2^ University Hospital Southampton NHS Foundation Trust, Southampton, United Kingdom; ^3^ University of British Columbia, Vancouver, BC, Canada

## Abstract

**Background:**

Medical advancements have led to an ageing population increasing the prevalence of Alzheimer's disease (AD), the most common form of dementia, poses a significant burden on the healthcare system. The diagnostic process is lengthy and complicated, averaging around 2 years. AD diagnosis is shifting from symptomatic assessment to physiological testing using fluid biomarkers and imaging. Neuroimaging techniques like Positron Emission Tomography (PET) measure amyloid load in dementia‐related brain regions. Artificial intelligence (AI) has shown potential for its application in PET scanning to identify patients with AD.

**Method:**

541 patients’ medical data was obtained from the Alzheimer's Disease Neuroimaging Initiative (ADNI) study, containing normal and cognitively impaired patients, were matched to their PET scans. Input for AI was based on the amyloid load on brain regions of interest obtained by PET. AI models were trained to classify patients into normal or abnormal for each biomarker threshold: amyloid PET (680pg/ml), tau (355pg/ml), and phosphorylated tau (56pg/ml), as defined by CSF measurements. Machine learning algorithms were trained and tested on up to 10 brain regions of interest for each biomarker, grouping patients into their disease states.

**Result:**

The *p*‐tau biomarker model achieved the highest test accuracy of 72.8% among all models. However, the amyloid‐beta (Aβ) biomarker model achieved better overall test metrics, with an area under the curve (AUC) of 0.80, indicating reliable classification performance. The precuneus and left lingual gyrus were significant regions for the Aβ model as per the Shapley importance values.

**Conclusion:**

Explainable AI shows potential in AD diagnosis from amyloid PET, achieving moderate accuracy using pathologically relevant brain regions. To increase accuracy and reduce dataset imbalances, a larger dataset and utilization of tools like stratified cross validation is necessary.

